# The association between social support and prosocial behavior: A three‐level meta‐analysis

**DOI:** 10.1002/pchj.792

**Published:** 2024-07-21

**Authors:** Yinlan Wang, Guangming Ran, Qi Zhang, Qiongzhi Zhang

**Affiliations:** ^1^ Department of Psychology, School of Education China West Normal University Nanchong China; ^2^ College of Preschool and Primary Education China West Normal University Nanchong China

**Keywords:** moderator variables, prosocial behavior, social support, three‐level meta‐analysis

## Abstract

Numerous studies have examined the relationship between social support and prosocial behavior and have concluded that social support is an important factor in generating prosocial behavior. However, different studies have produced different conclusions, and the moderating effect on the relationship is not entirely clear. The current study uses a three‐level meta‐analysis method to clarify the relationship between social support and prosocial behavior, and explores the moderating variables that affect the relationship between the two variables. Through a systematic literature search, a total of 92 studies, 418 effect sizes, and 74,378 participants were obtained. The main effects test found a significant positive correlation between social support and prosocial behavior. Tests of the moderating effects indicated that the relationship between social support and prosocial behavior was moderated by year of publication, source of social support, measurement of social support and measurement of prosocial behavior. In summary, social support plays an important role in prosocial behavior, and exploring their relationship is beneficial to families, schools and society in guiding individuals' prosocial behavior.

## INTRODUCTION

Positive psychology proposes that paying attention to people's positive power can also help people form a benign personality, so as to promote the healthy development of people and the harmonious development of the social environment (Seligman & Csikszentmihalyi, 2014). Prosocial behavior has been a hot research issue in the field of psychology. Generally speaking, it is the sum of behaviors that conform to social norms and expectations and are beneficial to others (Wittek & Bekkers, 2015; Zhou et al., 2014). Individuals with more prosocial behaviors are more likely to have higher life satisfaction, stronger self‐efficacy, and are less likely to have negative emotions such as depression and anxiety (Danielsen et al., 2010; Flynn et al., 2015; Weinstein & Ryan, 2010). Moreover, a growing body of research argues that social support is an important way for individuals to obtain positive emotions and help that promotes prosocial behavior (Lenzi et al., 2012; Li et al., 2019; Pfeiffer et al., 2016; Wang et al., 2022; Zhou & Feng, 2005). Broaden‐and‐build theory and social support theory both emphasize that the stronger the resources an individual possesses, the better they are able to cope with challenges from a variety of environments (Conway et al., 2013; Lakey & Cohen, 2000). Positive emotion resources expand one's way of thinking and behavioral shaping, enabling individuals to proactively engage in relevant activities in their growth and development, and providing sufficient resources for their thoughts and behaviors (Fredrickson, 2013). Therefore, the richer the moral support or material support an individual receives from the social relationships he or she has, the greater the likelihood of forming positive emotions. Prosocial behavior is more likely to occur in social helping situations (Telle & Pfister, 2016).

Prosocial behavior will not only affect individual's interpersonal communication and future development, but also promote social stability and harmony in the long run (Crone & Achterberg, 2022). The current study takes a novel approach of meta‐analyzing social support and prosocial behavior. From a statistical perspective, it explains the intrinsic mechanism between the two, and provides some realistic basis for the formation of future cultivation programs and policies. At the same time, it draws the attention of families, schools and society, and the tripartite coordination, to promote the more effective healthy development of individuals. Most of the previous studies are empirical and differ in terms of sample, publication status, and research tools. These uncertainties hinder researchers' understanding of the relationship between social support and prosocial behavior. The greatest advantage of three‐level meta‐analyses over traditional meta‐analyses is the ability to extract multiple effect sizes from a unified study, ensuring the integrity of the study (Konstantopoulos, 2011; Van den Noortgate et al., 2013). Accordingly, we used the three‐level meta‐analysis method to statistically synthesize previous studies to examine the relationship between social support and prosocial behavior. Moreover, which moderating effects may have an influence on the interpretation of the relationship between variables will also be explored.

### Conceptualization of social support and prosocial behavior

As is well known, interpersonal mutual support arose at the same time as human society. In the last century, social support began to be studied as a scientific subject of research in sociology, psychiatry and medicine (Gottlieb & Bergen, 2010). Social support was first introduced by sociologists and the concept entered the field of psychology with the emphasis on mental health (Taylor, 2011). However, according to existing research, the interpretation of social support has gone beyond its original scope, expanding to refer to the sum of social behaviors that provide individuals with mental and material resources to promote their better development (Bjørlykhaug et al., 2022; Malecki & Demaray, 2002). Therefore, this study considers social support to be the material or emotional help received by an individual, which can originate from the community, social network, or others (Cullen, 1994).

Categorized from the perspective of the source of social support, social support can be divided into family support, teacher support, peer support, and other support (Tao, 2000; Yin, 2023). There are some inconsistent findings in studies of social support from different sources. For example, in adolescents, peer support for individuals is more conducive to the generation of prosocial behaviors (Yang, 2022). Among college students, family support can have a significant effect on prosocial behavior (Pang, 2021).

Prosocial behavior refers to behaviors that are beneficial to others and society, and it is also called helpful behavior and helping behavior (Batson et al., 2011; Eisenberg & Fabes, 1998; Greener & Crick, 1999). It has been shown that prosocial behavior is not only influenced by some personality traits, but also by the social environment, and that groups with different cultural backgrounds and age characteristics behave differently regarding prosocial behavior (Orm et al., 2022; Stavrova et al., 2022). On the one hand, prosocial behavior can not only improve the interpersonal environment of individuals and promote the harmonious development of individuals, but also enhance the sense of self‐efficacy in this way (Campbell et al., 1999; Laible et al., 2004). On the other hand, prosocial behavior can create a good social environment, which is an important condition for the benign development of modern society (Kou & Tang, 2004). Therefore, the study of prosocial behavior is an important inspiration for the field of psychology concerning social adaptation behavior and individual socialization.

### Association between social support and prosocial behavior

Research has shown that prosocial behavior is closely related to individuals' perceived interpersonal and organizational affiliations (Cirelli et al., 2014), and that individuals are more likely to feel a sense of belonging and consider themselves part of a social environment when they are often helped by others in that environment, which is more conducive to prosocial behavior (Twenge et al., 2007). As a new motivation theory, self‐determination theory also confirms the relationship between social support and prosocial behavior. The degree of self‐determination in human behavior is influenced by internal motivation, and socially conditioned external support can enhance an individual's internal motivation by satisfying psychological needs (autonomy, support, competence), which can be externalized into behavior (Gagné et al., 2022; Guay, 2022). Specifically, individuals are more likely to exhibit prosocial behavior if they are in a social environment that is more socially supportive (De Guzman et al., 2012; Wei et al., 2017). According to reciprocal altruism (Trivers, 1971), altruistic behavior among people is reciprocal, which means that the social support an individual receives can have a significant impact on his or her altruistic behavior. Altruistic behavior is an internal experience and evaluation of the individual that is influenced by external support (Batson & Powell, 2003). Moreover, prosocial behavior contributes to good interpersonal relationships, which are the basis for social stability and a well‐developed social support system (Coulombe & Yates, 2022; Shao et al., 2023; Zhang & Zhao, 2021). In other words, there is a mutually reinforcing effect between these two variables.

In recent years, the relationship between social support and prosocial behavior has been receiving a lot of attention from the psychological community. While studies have confirmed a significant positive correlation between social support and prosocial behavior (Messman et al., 2022; Stanger et al., 2018), others have failed to explore this relationship (Yunanto, 2020). Vila (2021) and Zalta et al. (2021) focused on individuals being socially supported, while Luberto et al. (2018) and Memmott‐Elison et al. (2020) examined individuals' prosocial behaviors. These studies suggest that previous meta‐analyses have examined social support and prosocial behavior, but not both at the same time (Allen et al., 2021; Grey et al., 2020; Quarmley et al., 2022; Schindler & Friese, 2022; Thielmann et al., 2020). Therefore, using meta‐analysis and systematic reviews to clarify this uncertainty, a systematic integration of previous studies that have explored the relationship between social support and prosocial behavior is warranted.

### Impact of moderator variables

There have been several studies examining gender differences in social support, but no consistent conclusions have been drawn. Some studies have shown that women tend to achieve well‐being through codependent relationships, while men generally seek less social support and experience less psychological help than women (Burke & Kraut, 2016; Chu et al., 2010; Matud et al., 2003). A meta‐analysis also concluded that women were higher than men on all indicators of social support (Zhang et al., 2015). However, it has also been suggested that there are no gender differences in social support (Wen, 2021; Yang et al., 2022). Notably, there are also inconsistent findings on the gender effects of prosocial behavior. Studies have shown that women have a greater ability to understand the emotions and feelings of others than men, and thus show a higher propensity to help others (Murray et al., 2022), but a recent study suggests that men engage in more prosocial behavior during social dilemmas (Dorrough & Glöckner, 2021). Therefore, the current study explores whether gender might moderate the relationship between social support and prosocial behavior. It is also hypothesized that the correlation between social support and prosocial behavior is significantly higher in females than in males.

In addition, the age of the individual is a possible moderating variable. One study showed that the older an individual is, the greater the ability to perceive social support and therefore the higher the likelihood of prosocial behavior (Yang et al., 2017), with older adolescents being less socially active and receiving less information, with a corresponding decrease in the amount of social support received (Pfeil et al., 2009), which can affect prosocial behavior. Therefore, age was used as an important moderating variable in this meta‐analysis. It was also hypothesized that the relationship between the two variables would increase with age.

Cultural factors may be an important explanation for the controversy. The cultural context represents the physical and spiritual cultural environment that influences the physical and mental development and character formation of an individual. The social, economic, and cultural capitals held by Eastern and Western countries differ according to their social characteristics. In Eastern cultures, there is a greater emphasis on group orientation, and individuals are more considerate of the feelings of others in their behavior (Triandis, 2001). In the Western context, individual autonomy and peer competitiveness are more emphasized, and less prosocial behavior is likely to occur (Chen et al., 2006). Based on the above, the current study suggests that culture is a moderator of the relationship between the vocabulary of society and prosocial behavior, and hypothesizes that the correlation between social support and prosocial behavior is greater in the East.

There are many kinds of research designs in psychology. For example, a cross‐sectional study is a study in which individuals of different ages are selected at the same time, reflecting mainly inter‐individual differences. Longitudinal studies, on the other hand, are repeated studies with the same subjects over a considerable period of time and examine intra‐individual variation (Caruana et al., 2015; Niu & Ran, 2023). Furthermore, in a recent meta‐analysis on the prevalence of gaming disorders (Cheng et al., 2022), as well as in a study by Lam and Zhou (2022), it was noted that the study design could significantly modulate the relationship between variables. Therefore, the present study hypothesized that research hypothesis is a potential moderator.

Although total social support tends to be the most common variable analyzed, some scholars have suggested distinguishing between different sources of support in order to more accurately assess people's experience of support (Piko & Hamvai, 2010). According to ecological theory and as shown by Rodríguez‐Fernández et al. (2021), sources of social support can be categorized as family, teachers, and peers. Since most of the samples included in the study were students, teachers then become a source of social support that we cannot ignore. In addition, it is not possible that the three categories above are the only ones that have a close connection with the individual, so other people that have an important influence should also be the subject of our research (Zimet et al., 1988). In summary, the current study considers the source of social support to be a moderating variable in the relationship between social support and prosocial behavior. It is also hypothesized that teacher support will lead to an increase in the relationship between social support and prosocial behavior.

As we have seen, there are studies included in this study that date back to the last century. It has been suggested that the strength of the relationship between variables may change over time (Assink & Wibbelink, 2016; Hunsley & Meyer, 2003). Goulet and Cousineau's (2019) study pointed out that factors such as the passage of time and increasing the sample size enhance the statistical validity of the findings. Therefore, the present study proposes the hypothesis that an increase in the year of publication may result in an overall enhanced relationship between social support and prosocial behavior.

It is important to note that measures of prosocial behavior may also be a factor in moderating the relationship between social support and prosocial behavior (Carlo et al., 2010; Martí‐Vilar et al., 2019). The literature included in this meta‐analysis has a great variety of instruments for measuring prosocial behavior, and the division of dimensions between questionnaires, with significant differences in statistical methods, may have influenced the results of the study. Studies have shown that the differences between variables will vary depending on the measurement tools (Anderson et al., 2010; Xu et al., 2021). Thus, we hypothesized that the instrument measuring prosocial behavior might be a moderating variable between social support and prosocial behavior.

### Current research

The results obtained from empirical studies on social support and prosocial behavior to date have been inconsistent (Rupika et al., 2017), and no studies have conducted a comprehensive quantitative analysis of the association between these two variables. Moreover, the findings and investigative focus of previous studies differ due to differences in sample, publication characteristics, study design, and evaluation. Therefore, this meta‐analysis serves two purposes: (1) to examine the overall relationship between social support and prosocial behavior; and (2) to explore which factors may further influence the overall correlation between the two variables by performing a moderating effect analysis.

## METHODS

The Preferred Reporting Items for Systematic Evaluation and Meta‐Analysis (PRISMA) can be used as reporting guidelines through which meta‐analysis reporting can be improved and standardized (Moher et al., 2015). Moreover, to increase transparency and avoid wastage of research resources, this meta‐analysis has been registered in the International Prospective Register for Systematic Reviews (PROSPERO; Registration number: CRD42023393307; URL: https://www.crd.york.ac.uk/prospero/; Lakens et al., 2016).

### Data source and inclusion guidelines

We searched PsycINFO, PubMed, ScienceDirect, Google Scholar (GS) and China National Knowledge Infrastructure (CNKI) up to January 2023. The literature search was done by two authors (G.R. and Y.W.) alone in separate time periods. The research strategy was developed jointly by the authors and two peer researchers, without language restrictions.

To determine the search terms, we looked up words related to the study topic in the dictionary, and the keywords that occurred most frequently were summarized by two authors (G.R. and Y.W.). The search for this study consisted of these two main components: (a) social support and (b) prosocial behavior. As displayed in Table 1, for social support, the main keywords used in the search were “support” OR “social support.” For prosocial behavior, the main keywords used in the search were: “prosocial behavior” OR “pro‐social behavior” OR “prosocial behaviors” OR “helping behavior” OR “altruistic behavior” OR “altruism.” We searched the database for keywords for these two elements in the title, abstract and keywords to obtain the relevant studies. Moreover, we also conducted a screening for a more comprehensive access to the relevant literature (i.e., manual searches of relevant journals, forward searching, and backward searches).

**TABLE 1:** Key words of two search elements.

Search elements
(a) Social support: “support” OR “social support” (b) Prosocial behavior: “prosocial behavior” OR “pro‐social behavior” OR “prosocial behaviors” OR “helping behavior” OR “altruistic behavior” OR “altruism” OR “altruistic behavior”

**FIGURE 1:**
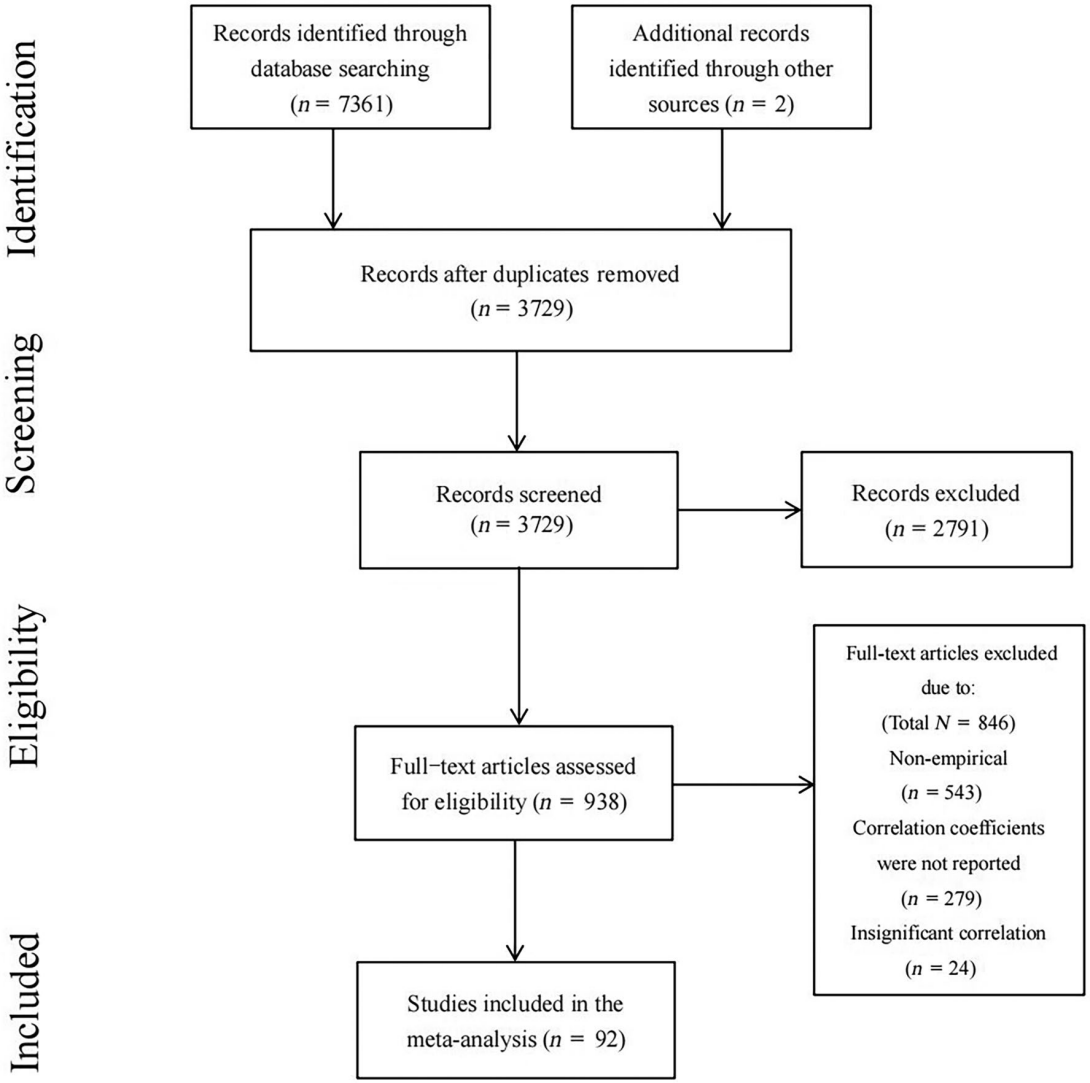
The PRISMA flow chart used to identify studies for detailed analysis of social support and prosocial behavior.

For the retrieved literature, this meta‐analysis was further screened using the following criteria: (a) the study was an empirical study and not a review article; (b) objective instruments were used to measure the relationship between social support and prosocial behavior; (c) the article was a longitudinal study or a cross‐sectional study; and (d) at least one correlation coefficient was reported for social support (different dimensions of the scale or different sources of social support) and prosocial behavior, or statistics were shown that could be converted to a correlation coefficient; (e) when duplicate datasets were available, articles published in academic journals using the same dataset were included. Ultimately, a total of 92 studies were included in this meta‐analysis, and the selection process is shown in Figure 1.

### Literature quality evaluation

We followed the criteria for assessing the quality of literature compiled by Zhang et al. (2019) in the relevant meta‐analysis study. The criteria assessed the quality of the included literature in terms of subject selection, data validity, validity of measurement tools, and publication level. The final total score for each study was calculated between 0 to 10, with higher scores indicating better quality literature (see Table 2).

**TABLE 2:** Characteristics of the 92 studies included in the meta‐analysis.

First author (year)	K	*N*	Gender	Age	Culture	*SS* scale	*PB* scale	Publication status	Literature quality
Ban and Song (2012)	18	1576	0.46	C	E	SSRS	PTM	Published	8
Bao (2021)	1	502	0.40	Ad	E	OSSQ	IABSU	Unpublished	4
Bao (2022)	1	394	0.47	A	E	MSPSS	IABSU	Unpublished	8
Chang et al. (2021)	3	1984	0.44	Ad	E	Other	Other	Published	8
Che (2020)	28	416	0.60	Ad	E	SSRS	PTM	Unpublished	4
Chen (2017)	27	221	0.53	Ad	E	MSPSS	PTM	Unpublished	5
Chen (2020)	25	495	0.59	A	E	SSRS	PTM	Unpublished	6
Chen and Feng (2022)	1	214	‐	C	E	SSRS	Other	Published	7
Cheng (2016)	4	313	0.49	C	E	SSRS	PTM	Unpublished	5
Crosby III and Smith (2015)	1	279	‐	C	W	Other	Other	Published	8
Dang (2020)	1	566	0.48	Ad	E	Other	PTM	Unpublished	6
Fu, Feng, et al. (2022)	3	1171	0.47	Ad	E	Other	Other	Published	10
Fu, Wang, et al. (2022)	1	676	0.25	A	E	MSPSS	PTM	Published	9
Griese and Buhs (2014)	3	511	0.49	C	W	Other	Other	Published	9
Guo et al. (2014)	1	260	0.47	C	E	MSPSS	SDQ	Published	6
Guo (2014)	16	432	0.41	C	E	SSRS	PTM	Unpublished	6
Guo (2018)	4	720	0.54	A	E	SSRS	PTM	Published	8
Guo (2020)	2	570	0.27	A	E	OSSQ	IABSU Other	Unpublished	6
Guo (2022)	1	691	0.36	A	E	MSPSS	PTM	Published	7
Ha (2004)	2	320	0.54	Ad	E	Other	Other	Published	8
Hastings (2003)	1	78	–	C	W	Other	SDQ	Published	9
Hu (2019)	6	773	0.52	Ad	E	CASSS	PTM	Unpublished	8
Hu et al. (2022)	1	2989	0.30	A	E	MSPSS	PTM	Published	9
Huang (2018)	1	700	0.42	A	E	OSSQ	IABSU	Unpublished	7
Huang (2020)	1	1147	0.57	C	E	SSRS	Other	Published	5
Huang et al. (2021)	1	636	0.49	A	E	MSPSS	PTM	Published	7
Jang et al. (2020)	1	1006	0.45	Ad	E	Other	Other	Published	8
Jiang et al. (2016)	1	392	0.35	A	E	Other	IABSU	Published	6
Jin et al. (2021)	1	256	0.30	A	E	Other	Other	Published	9
Lenzi et al. (2012)	2	1145	1.00	C	W	MSPSS	Other	Published	7
Li, Jiang, et al. (2018)	1	328	0.45	A	E	Other	Other	Published	7
Li, Zhao, et al. (2018)	1	513	0.19	A	E	MSPSS	Other	Published	8
Li, Zhou, and Xu (2018)	1	287	0.46	A	E	OSSQ	IABSU	Published	8
Li et al. (2019)	18	900	0.48	Ad	E	SSRS	PTM	Published	9
Li et al. (2020)	1	512	0.47	Ad	E	CASSS	Other	Published	6
Li et al. (2023)	1	858	0.41	Ad	E	MSPSS	Other	Unpublished	5
Liang (2015)	1	300	–	A	E	SSRS	Other	Unpublished	5
Liang (2020a)	2	1064	0.39	A	E	OSSQ	IABSU	Unpublished	7
Liang (2020b)	28	401	0.47	Ad	E	SSRS	PTM	Unpublished	6
Liu et al. (2016)	1	491	0.45	A	E	OSSQ	IABSU	Published	10
Liu, Wu, et al. (2021)	1	1995	0.41	A	E	SSRS	Other	Published	9
Liu, Wang, and Wu (2021)	1	492	‐	Ad	E	Other	PTM	Published	8
Lu (2021)	1	320	0.49	Ad	E	MSPSS	PTM	Unpublished	8
Lu (2022)	1	1701	0.42	Ad	E	MSPSS	PTM	Published	9
Messman et al. (2022)	2	837	0.50	Ad	W	Other	SDQ	Published	6
Oh and Roh (2022)	1	480	0.67	A	E	Other	Other	Published	6
Pang (2021)	28	1037	0.48	A	E	MSPSS	PTM	Unpublished	6
Qin et al. (2016)	3	1573	0.46	Ad	E	SSRS	SDQ	Published	5
Qiu and An (2012)	3	271	0.55	Ad	E	Other	SDQ	Published	7
Shao and Hu (2018)	1	417	0.53	A	E	SSRS	Other	Published	8
Simões et al. (2018)	3	818	0.46	Ad	W	Other	PTM	Published	6
Smith and Crosby III (2021)	1	612	0.45	C	W	Other	Other	Published	4
Stanger et al. (2018)	2	275	0.55	Ad	W	Other	Other	Published	6
Su and Wang (2022)	1	1007	0.67	A	E	MSPSS	Other	Published	10
Teng and Zhang (2022)	1	984	0.54	A	E	OSSQ	Other	Published	9
Tian (2011)	14	130	0.51	A	E	MSPSS	PTM	Unpublished	7
Tian (2020)	1	510	0.51	C	E	Other	Other	Unpublished	8
Wang (2011)	4	589	0.57	Ad	E	Other	PTM	Published	8
Wang and Wu (2020)	1	542	0.46	Ad	E	Other	PTM	Published	7
Wang (2021)	28	495	0.51	C	E	SSRS	PTM	Unpublished	8
Wei (2019)	1	783	0.46	A	E	MSPSS	PTM	Unpublished	7
Wen (2021)	1	641	0.48	Ad	E	MSPSS	PTM	Unpublished	8
Wentzel and McNamara (1999)	1	167	0.51	C	W	Other	Other	Published	5
Wong et al. (2017)	1	213	0.27	Ad	E	Other	Other	Published	8
Wu (2020)	2	1112	0.28	A	E	SSRS	IABSU	Unpublished	8
Xu et al. (2022)	4	423	0.29	A	E	MSPSS	Other	Published	8
Xue et al. (2022)	1	313	0.40	A	E	SSRS	PTM	Published	9
Yang et al. (2017)	1	442	0.40	A	E	OSSQ	IABSU	Published	8
Yang (2020)	1	600	0.38	Ad	E	CASSS	PTM	Unpublished	8
Yang (2022)	28	818	0.51	Ad	E	MSPSS	PTM	Unpublished	7
Yang et al. (2022)	1	581	0.38	Ad	E	CASSS	PTM	Published	7
Ye et al. (2020)	3	970	0.41	A	E	MSPSS	Other	Published	6
You et al. (2020)	1	8877	0.50	C	E	Other	Other	Published	7
Zeng (2012)	4	694	0.48	A	E	MSPSS	Other	Published	5
Zhang et al. (2021)	1	3346	0.45	A	E	MSPSS	PTM	Published	6
Zhang, Shen, and Hu (2022)	2	773	0.52	Ad	E	CASSS	PTM	Published	7
Zhang, Deng, et al. (2022)	1	1026	0.29	A	E	SSRS	PTM	Published	8
Zhao et al. (2012)	1	560	0.40	A	E	OSSQ	IABSU	Published	9
Zhao (2017)	4	309	0.43	Ad	E	SSRS	SDQ	Published	4
Zhao et al. (2017)	1	1238	0.44	Ad	E	MSPSS	PTM	Published	5
Zhao (2020)	28	324	0.42	Ad	E	CASSS	PTM	Unpublished	7
Zhao et al. (2020)	1	714	0.50	C	E	MSPSS	Other	Published	8
Zheng (2012)	1	496	0.55	A	E	OSSQ	IABSU	Published	9
Zheng (2013)	2	366	0.45	A	E	OSSQ	IABSU	Published	9
Zheng et al. (2017)	1	887	0.42	Ad	E	OSSQ	IABSU	Published	8
Zheng (2017)	1	970	0.41	A	E	OSSQ	Other	Unpublished	8
Zheng (2021)	1	841	0.41	Ad	E	MSPSS	PTM	Unpublished	8
Zheng et al. (2021)	1	813	0.32	A	E	OSSQ	IABSU	Published	9
Zhong (2020)	2	861	0.39	A	E	OSSQ	IABSU	Unpublished	7
Zhou (2012)	1	441	0.50	A	E	SSRS	Other	Unpublished	5
Zhou (2022)	1	543	0.52	Ad	E	SSRS	PTM	Unpublished	6
Zhu et al. (2022)	4	1064	0.39	A	E	OSSQ	IABSU	Published	8

*Note*: A, adults; Ad, adolescents; C, Children; CASSS, Child and Adolescent Social Support Scale; E, Eastern; Gender, percentage of males; K, number of effect sizes; MSPSS, Multidimensional Scale of Perceived Social Support; N, number of participants; OSSQ, Online Social Support Questionnaire; PB, prosocial behavior; SS, social support; SSRS, Social Support Rating Scale; W, Western; For prosocial behavior scale: IABSU, Internet Altruistic Behavior Scale for College Students; PTM, Prosocial Tendencies Measure; SDQ, Strengths and Difficulties Questionnaire.

### Coding of studies

In order to conduct a valid quantitative analysis of the included literature, this study followed the method of Lipsey and Wilson (2001) for coding the literature data: article title, publication year, first author's name, sample size, mean age, male proportion, and effect size. Moreover, we can divide this study into three parts: sample characteristics, publication characteristics, and evaluation characteristics based on previous experience (Ran, Zhang, et al., 2022).

The effect sizes in the coding are all derived from independent samples. Therefore, when multiple independent samples are reported in a study, we should code accordingly. In addition, as the effect size comes from different studies, it is easy for coding errors to occur. In order to avoid this problem, all initial documents were recoded independently by two researchers (G.R. and Y.W.) in two time periods (1 month apart), based on the methodology used in previous studies. The values for the sample characteristics were: gender (intraclass correlation coefficient [ICC] = 0.999), age (*k* = 1.000), and culture (ICC = 1.000); for the publication characteristics: year of publication (ICC = 0.998) and publication status (*k* = 1.000); and for assessment and study design characteristics: type of social support (*k* = 1.000), social support measure (*k* = 0.990), and prosocial behavior measure (*k* = 0.967). Typically, ICC is judged as good agreement between 0.40 and 0.75, and above 0.75 indicates high agreement. Cohen's kappa (*k*) are judged as good agreement between 0.40 and 0.59, 0.60–0.74 is fairly good, and 0.70 and above is very good agreement (Orwin, 1994). Therefore, the agreement between the two researchers in this study reached a high level.

### Sample characteristics

This meta‐analysis includes three sample features. First, the proportion of men was coded. Second, the growth stage of the participants was divided into three parts: children from 0 to 11 years old, adolescents from 12 to 18 years old and adults over 18 years old, according to the age classification in the field of medicine (Pearson & Biddle, 2011). In addition, for some studies in which the age of the participants was unclear, we performed the following classification: (a) elementary school students are coded as children; (b) middle school and high school students coded as adolescents; (c) college students were coded as adults. Finally, the study participants were coded as Eastern or Western according to their national cultures (Chang et al., 2011).

### Publication characteristics

Publication characteristics include the following two types: year of publication and publication status: (a) the year of publication is coded as a continuous variable; (b) the publication status includes published and unpublished. Published means published in academic journals, and unpublished mainly includes academic papers and conference papers.

### Assessment and research design characteristics

Literature quality assessment was performed using the Quality Assessment Tool for Observational Cohort and Cross‐Sectional Studies (Nih et al., 2018). For research design characteristic, instruments to assess social support and prosocial behavior were coded. Based on the available research, it is known that there are a variety of instruments used to assess social support and prosocial behavior. Therefore, we primarily created five social support assessment categories (OSSQ vs. SSRS vs. MSPSS vs. CASSS vs. Others) and four prosocial behavior assessment categories (IABSU vs. PTM vs. SDQ vs. Others).

### Data analysis

We retrieved a large number of studies exploring the relationship between social support and prosocial behavior. We selected the correlation coefficient (*r*) as the effect size before performing the meta‐analysis, and converted the correlation coefficient into normal Fisher's *Z*‐scores, and then the Fisher's *Z*‐scores were converted back to correlation coefficients to facilitate interpretation of the results (Hedges & Olkin, 1985). As previously noted, some of the literature for this study contains multiple effect sizes. However, traditional univariate meta‐analysis requires each effect size to be independent of each other, which means that each study can only include one effect size. This is because multiple effect sizes originating from the same study can exaggerate the correlation between variables. Recent research has shown that three‐level meta‐analysis allows a study to incorporate multiple effect sizes when considering dependence (Cheung, 2014). Therefore, we used a three‐level random effects model to estimate the association between social support and prosocial behavior with moderator analysis.

Previous studies have shown that the three‐level meta‐analysis model divides the sources of variance into sampling variance (level 1), within‐study variance (level 2), and between‐study variance (level 3) (Yang et al., 2021). The three‐level meta‐analysis in this study was allowed to retain all effect sizes from the original literature, which is different from the classical meta‐analytic model, and this approach enables the maximization of statistical functional rates (Assink et al., 2015). In this study, Version 4.0.3‐win of the R software was used in the data analysis version of the metafor package for meta‐analysis. Model guidelines for multilevel random effects were developed in accordance with the tutorial of the metafor package (Viechtbauer, 2010).

Publication bias is the greater likelihood that statistically significant study results will be reported and published than non‐significant and invalid results (Franco et al., 2014). When publication bias is a more serious problem, it means that the results and conclusions of the meta‐analysis are not sufficiently valid (Thornton & Lee, 2000). This study includes published research and academic papers, and we try to include all eligible literature to reduce the impact of publication bias on the study results. In this study, the funnel plots and Egger's regression tests were used to estimate publication bias (Rothstein et al., 2006). The researcher observes that if the shape of the funnel plot is presented symmetrically, the publication bias is negligible. The result of the Egger's regression method is not significant, indicating that there is no significant publication bias (Egger et al., 1997), and if the number of missing studies was larger than Rosenthal's criterion (5*k* + 10, where *k* is the number of effect sizes) (Li et al., 2021), the publication bias can also be ignored. When there are significant publication deviations, the adjustment is made by the trim‐and‐fill method (Duval & Tweedie, 2000).

## RESULTS

### Study characteristics and quality assessment

Based on the systematic literature search described above, this meta‐analysis retrieved a total of 92 eligible papers, including 418 effect sizes and 74,378 participants (shown in Table 2). The minimum sample size is 78 and the maximum is 8877, and the mean age of the participants was 15.41 years. Publication years span from 1999 to 2023. The minimum number of effect sizes in the included literature was 1 and the maximum was 28. Two forms of moderating variables were included: continuous variables and categorical variables. The effect sizes for continuous moderating variables were 413 (gender) and 418 (year of publication), and the number of effect sizes for categorical variables were as follows: age (children: 81; adolescents: 212; adults: 125), publication status (published: 136; unpublished: 282), culture (Western: 16; Eastern: 401); sources of social support (family: 44; peer: 40; teacher: 13; other: 23); measures of social support (OSSQ: 24; SSRS: 184; PSSS: 127; CASSS: 39; other: 44); measures of prosocial behavior (PTM: 326; IABSU: 24; SDQ: 16; other: 52). The mean quality score of the included literature was 7.18, with four of the studies scoring below the theoretical mean of 5.

### Publication bias

**FIGURE 2:**
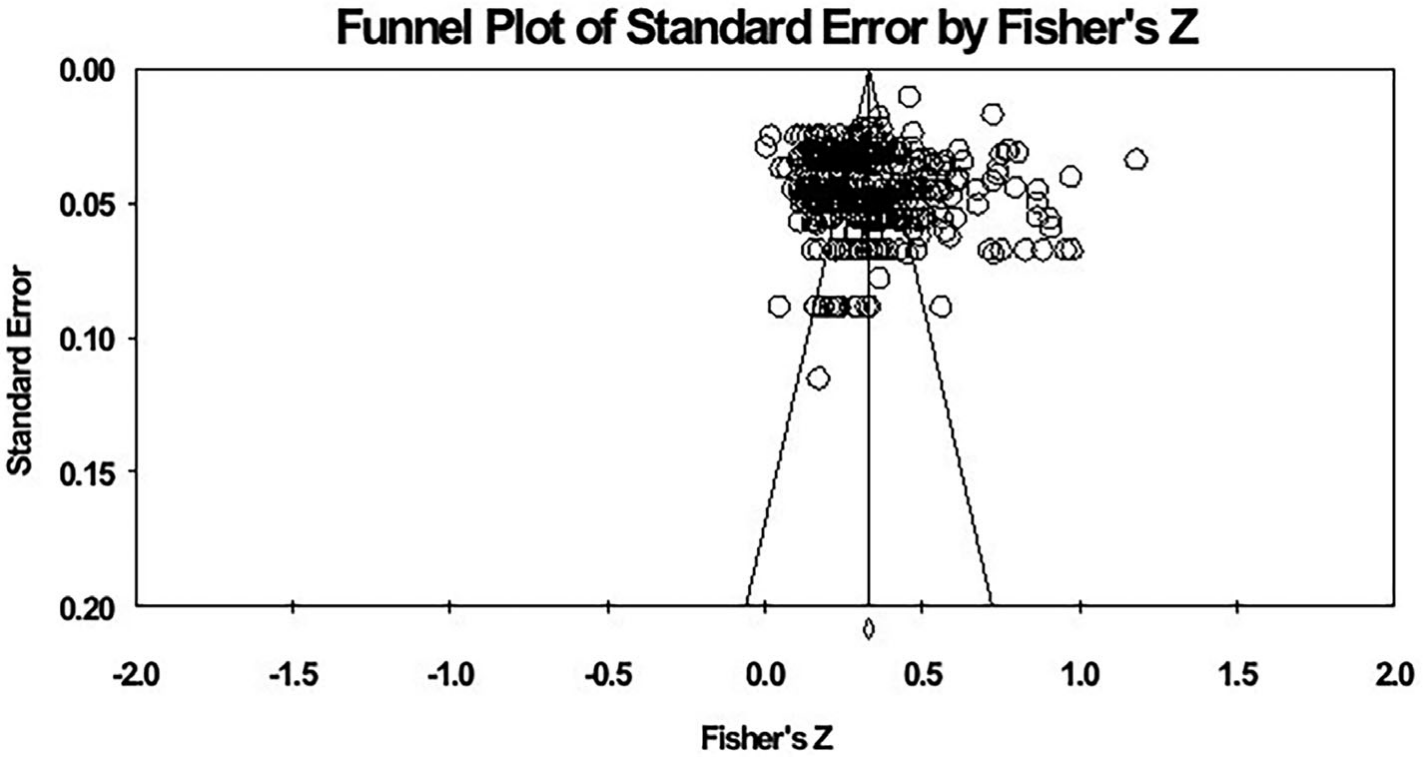
Funnel plot of effect sizes of the correlation between social support and prosocial behavior.

Inspection of the funnel plot reveals that the points representing the effect sizes on the plot are roughly evenly distributed on both sides of the mean effect size and are located at the top (see Figure 2), which indicates that there is no publication bias in this study (Duval & Tweedie, 2000). Another test was performed using *Fail‐safe* N and Egger's linear regression (Rosenthal, 1979). The test yielded a fail‐safe coefficient of 2,870,165, both greater than the critical value of 2100, which is 5*k* + 10. We also performed an Egger's test, which yielded a non‐significant *p*‐value (*t* = 0.562, *p = *.575), again indicating that there was no significant publication bias in this meta‐analysis. As the funnel plot and Egger's test both showed that the publication bias was not significant, we did not perform further publication bias tests for this meta‐analysis using Tweedie's trim‐and‐fill analysis (Ran, Li, et al., 2022).

### Sensitivity test

In order to measure the interference of extreme values in individual studies with the results of the meta‐analysis, we performed sensitivity analyses by progressively excluding each study from review. The results showed fluctuations in social support and prosocial behavior effect sizes ranging from 0.317 to 0.320, which is close to the total effect size of 0.318, indicating that the literature included in the meta‐analysis is not influenced by extreme values and the results are stable.

### Overall effect sizes

In this study, a random effects model was used to test the main effects of the relationship between social support and prosocial behavior (see Table 3). The results showed a significant positive correlation between social support and prosocial behavior (*r* = 0.409, *p < *.001). Also, the log‐likelihood‐ratio test showed significance between the variables (*p < *.001) in both within‐study variance (level 2) and between‐study variance (level 3). Of the total sources of variance, the sampling variance (level 1) was 3.46%, the within‐study variance (level 2) was 22.77%, and the between‐study variance (level 3) was 73.77%. This distribution indicates that there are moderating variables at play between the two variables (Ran, Zhang, et al., 2022). Therefore, this study can analyze the moderating variables in order to further explain the relationship between social support and prosocial behavior.

**TABLE 3:** Results of a random effects model of the relationship between social support and prosocial behavior.

Social support	*k*	Fisher*'Z* (*SE*)	95% CI	*t*	*r*	Level 1 (%)	Levels 2 variance	Levels 3 variance
							Levels 2 (%)	Levels 3 (%)
Prosocial behavior	418	0.434 (0.021)	[0.392, 0.475]	20.709	0.409	3.46%	0.010***	0.032***
							22.77%	73.77%

Note: *k* = number of effect sizes; *SE* = standard error; 95% CI = 95% confidence interval; Levels 2 variance = variance between effect sizes extracted from the same study; Levels 3 variance = variance between studies; Level 1 (%) = Level 1 as a percentage of total variants; Level 2 (%) = Level 2 as a percentage of total variants; Level 3 (%) = Level 3 as a percentage of total variants.

*** *p < *.001.

### Moderator analyses

We produce the results of the moderators of the relationship between social support and prosocial behavior in Table 4. The results of the study showed that publication year was a significant moderating variable (*F* (1, 416) = 6.579, *p = *.011). In other words, the relationship between these two variables has been higher in intensity in recent years. In addition, the source of social support was a significant moderating variable (*F* (3,115) = 2.870, *p = *.040). We found that support from teachers (*r* = 0.400) had a greater effect on prosocial behavior than from family (*r* = 0.353), peers (*r* = 0.367) and others (*r* = 0.384). Notably, the measure of social support (*F* (4,413) = 5.270, *p < *.001) was also found to have a moderating effect, with stronger associations between social support and prosocial behavior found using the OSSQ (*r* = 0.508) measure than using the SSRS (*r* = 0.310) measure. More importantly, the measure of prosocial behavior (*F* (3, 414) = 5.734, *p < *.001) also had a moderating effect, especially when measured using the IABSU (*r* = 0.536). Finally, gender (*F* (1, 411) = 2.633, *p = *.105), age (*F* (2, 415) = 1.930, *p = *.146), culture (*F* (1, 416) = 0. 140, *p = *.170), study design (*F* (1, 416) = 1.851, *p = *.174) and publication status (*F* (1, 416) = 0.159, *p = *.690) were not found to have a moderating effect in the current study.

**TABLE 4:** Results of categorical and continuous moderators for the association between social support and prosocial behavior.

#Moderator variables	#ES	Intercept/mean *z* (95% CI)	*β*_1_ (95% CI)	Mean *r*	*F*(df1, df2)^a^	*p* ^b^	Levels 2 variance	Levels 3 variance
**(1) Sample characteristics**								
a. Gender	413	0.433 (0.386; 0.476)***	−0.159 (−0.351; 0.034)	–	*F* (1, 411) = 2.633	0.105	0.010***	0.033***
b. Age								
Children	81	0.437 (0.339; 0.534)***		0.411	*F* (2, 415) = 1.930	0.146	0.010***	0.031***
Adolescents	212	0.385 (0.320; 0.450)***	−0.052 (−0.069; 0.065)	0.367				
Adults	125	0.473 (0.413; 0.534)***	0.036 (−0.078; 0.151)	0.441				
c. Culture								
Eastern	402	0.436 (0.393; 0.479)***		0.410	*F* (1, 416) = 0.140	0.708	0.010***	0.032***
Western	16	0.409 (0.276; 0.543)***	−0.027 (−0.167; 0.114)	0.388				
**(2) Publication characteristics**								
a. Publication year	418	0.430 (0.390; 0.470)***	0.012 (0.003; 0.022)*	–	*F* (1, 416) = 6.579	0.011	0.010***	0.030***
b. Publication status								
Published	136	0.440 (0.389; 0.490)***		0.414	*F* (1, 416) = 0.159	0.690	0.010***	0.032***
Unpublished	282	0.422 (0.352; 0.492)***	−0.018 (−0.104; 0.069)	0.399				
**(3) Assessment and research design characteristics**								
a. Design								
Cross	408	0.427 (0.385;0.468)***		0.403	*F* (1, 416) = 1.851	0.174	0.010***	0.032***
Long	10	0.550 (0.377;0.722)***	0.123 (−0.055; 0.301)	0.501				
b. Sources of social support								
Family	44	0.369 (0.258; 0.479)***		0.353	*F* (3, 115) = 2.870	0.040	0.002***	0.056***
Peer	40	0.385 (0.275; 0.495)***	0.016 (−0.014; 0.046)	0.367				
Teacher	13	0.424 (0.308; 0.539)***	0.055 (0.007; 0.103)*	0.400				
Other	23	0.405 (0.292; 0.517)***	0.036 (0.002; 0.070)*	0.384				
c. Measurement of social support								
OSSQ	24	0.560 (0.474; 0.646)***		0.508	*F* (4, 413) = 5.270	<0.001	0.010***	0.024***
SSRS	184	0.320 (0.253; 0.388)***	−0.240 (−0.346; −0.133)***	0.310				
MSPSS	127	0.443 (0.372; 0.513)***	−0.117 (−0.228; −0.006)*	0.416				
CASSS	39	0.492 (0.352; 0.634)***	−0.067 (−0.232; 0.098)	0.456				
Other	44	0.416 (0.348; 0.484)***	−0.144 (−0.252; −0.035)**	0.394				
d. Neasurement of prosocial behavior								
PTM	326	0.393 (0.333; 0.453)***		0.374	*F* (3, 414) = 5.734	<0.001	0.010***	0.029***
IABSU	24	0.598 (0.507; 0.688)***	0.205 (0.096; 0.314)***	0.536				
SDQ	16	0.328 (0.202; 0.454)***	−0.065 (−0.200; 0.071)	0.317				
Other	52	0.421 (0.356; 0.487)***	0.029 (−0.060; 0.118)	0.398				

*Note*: #ES = number of effect sizes; mean *z* = mean effect size (Fisher's *z*); 95% CI = 95% confidence interval.

Abbreviations: df, degrees of freedom; Levels 2 variance, variance between effect sizes extracted from the same study; Levels 3 variance, variance between studies; *r*, mean effect size expressed as a Pearson's correlation; β_1_, estimated regression coefficient; For social support scale: CASSS, Child and Adolescent Social Support Scale; MSPSS, Multidimensional Scale of Perceived Social Support; OSSQ, Online Social Support Questionnaire; SSRS, Social Support Rating Scale; For prosocial behavior scale: IABSU, Internet Altruistic Behavior Scale for College Students; PTM, Prosocial Tendencies Measure; SDQ, Strengths and Difficulties Questionnaire.

^a^ Omnibus test of all regression coefficients in the model.

^b^ *p*‐value of the omnibus test.

* *p < *.05;

** *p < *.01;

*** *p < *.001.

## DISCUSSION

Research has shown that prosocial behavior can lead individuals to produce more positive emotions and less negative emotions (Miles et al., 2022). A large number of empirical studies have confirmed that social support and prosocial behavior are related (Chen, 2020; Hu et al., 2022). However, there is uncertainty about the relationship between social support and prosocial behavior. Considering this issue, the three‐level meta‐analysis was used to explore the relationship between these two variables.

A total of 92 studies, 418 effect sizes and 74,378 participants were included in this study. The results of the three‐level meta‐analysis confirmed a strong relationship between social support and prosocial behavior, which indicates that individuals who received more social support were more likely to exhibit prosocial behavior. Moreover, the strength of this relationship was influenced by publication year, the source of social support, the measure of social support, and the measure of prosocial behavior, meaning that the moderating variables mentioned above played an important role in the relationship between social support and prosocial behavior. This study provides a basis for further explaining the relationship between social support and prosocial behavior and initiatives to promote prosocial behavior.

### Overall relationship

The present study clarifies the debate that exists regarding the magnitude of the correlation between social support and prosocial behavior and supports the majority of the current findings. The results showed a significant positive correlation between social support and prosocial behavior. When individuals have more social support, they show high levels of prosocial behavior (Jin et al., 2021; Shao & Hu, 2018; Smith & Crosby III, 2021). Social cognitive theory suggests that individuals form inferences about others and things based on social information, which can have an impact on the emergence of behavior (Beauchamp et al., 2019; Schunk & DiBenedetto, 2020). The more social support an individual has, the more supportive resources he or she perceives, which provides a good environment for promoting prosocial behavior (Greitemeyer, 2022; Paulus, 2018). Moreover, when individuals perceive a cordial interpersonal environment and close organizational relationships, they will be prompted to produce positive behaviors, which is consistent with the results of this study. The more support they feel from various parties, the more willing they are to provide help to those in need and try to give back to society, which will stimulate more prosocial behaviors (Liu et al., 2016; Xu et al., 2022).

In addition, this relationship can be explained by social exchange theory, which advocates explaining social behavior in terms of input–output relationships in economics (Cropanzano & Mitchell, 2005). In other words, the principle of human society is to help each other, and when an individual feels more social support, the greater the sense of benefit, the more inclined he or she is to help others, and the frequency of prosocial behavior is higher (Cropanzano et al., 2017).

### Explaining heterogeneity with moderators

Notably, publication year significantly moderates the relationship between social support and prosocial behavior, as shown by the characteristics of the literature. The collected literature was published between 1999 and 2023, at the turn of the century, when society was developing at a high pace and living conditions were getting better—for instance, the development of networks and community clusters (Suanet & Antonucci, 2017). It has been noted that the interactive and immediate nature of online social support greatly facilitates the development of an individual's mental health (Greenhow et al., 2022; Zhou & Cheng, 2022). Examples include online counseling and sharing of educational resources, which were not available in previous years (Le et al., 2023; Tlili et al., 2023). This means that the types of social support available to people are more diverse and increasing in number (House et al., 1988; LaCoursiere, 2001), creating favorable conditions for prosocial behaviors (Agneessens et al., 2006; Corritore et al., 2003).

Moreover, the current findings suggest that the sources of social support are significant moderating factors. Although support from teachers, family, peers, and others all influenced the onset of prosocial behavior, support from teachers was slightly higher than other sources of support, suggesting that support from teachers may be more helpful to students in developing prosocial behavior. This may be because most of the participants in this study were students (Liu, Wang, & Wu, 2021; You et al., 2020; Zhang et al., 2021). Support from outside the family seems to be increasingly important as individuals grow older and expand their range of interactions (Schacter & Margolin, 2019). School is the most important site of activity other than the home, and although students' sense of individuality is gradually awakening, mindsets and behavioral norms are influenced by obedience to authority and academic achievement (Wang, 2011). According to attachment theory (Johnson, 2019), from the time of becoming a student, an individual's attachment is not limited only to parents, but in some cases, teachers provide security at school and are important attachment objects in addition to parents (Spivak & Farran, 2012; Verschueren & Koomen, 2012). Therefore, teacher support has a significant impact on an individual's behavior (Zhang, Shen, & Hu, 2022). This suggests that, as stated by ecosystem theory (Bronfenbrenner, 1992), the microsystems that act directly on individuals, such as family, peers, and school, have the greatest influence on their psychological development and behavioral performance at different stages of their development. The average age of the participants in this meta‐analysis was about 15 years old, when the individual is usually a student, so the support of teachers in school was important to the individual.

Moreover, with regard to measurement factors, the results of the meta‐analysis indicated that measures of social support moderated significantly the positive correlations between social support and prosocial behavior. This means that different instruments for measuring social support affect the degree of correlation between social support and prosocial behavior. Specifically, the correlation between the variables was greatest when the instrument measuring social support was the OSSQ, and the correlation between the two was smallest when measured with the CASSS. The current study suggests that the main reason for this is the difference in the measurement tool divided into dimensions. The OSSQ contains four dimensions of emotional support, instrumental support, informational support, and peer support, and the CASSS is divided into subscales from parents, teachers, classmates, close friends, and school (Liang, 2020a; Luo et al., 2017). The OSSQ also focuses more on examining social support from the Internet (Liang & Wei, 2008; Turner et al., 2001). Therefore, the higher degree of correlation when measured using the OSSQ is strongly related to the greater specificity of this measurement tool.

Furthermore, it is worth noting that the results of this study also showed a significant moderating effect of the prosocial behavior measure. As the findings suggest, IABSU plays a significant regulating role as a measurement tool, possibly because IABSU is a measure of online altruism, and people are more willing to help others online than in the real world, with more online altruism than real altruism (Wallace, 2015). This suggests that we should probably be careful in the choice of measurement instruments in further analyses and follow‐up studies.

### Implications

This study integrates empirical research on social support and prosocial behavior using three‐level meta‐analytic techniques to explore the relationship and its moderating variables. First, this study found that publication year moderated the relationship between social support and prosocial behavior. This suggests that the diversity of social support provides favorable conditions for the emergence of prosocial behaviors as time progresses. Second, this study found that teacher support and other people's support among the sources of social support moderated the relationship between social support and prosocial behavior. This suggests that teachers need to be aware of their own words and behaviors, which may have a significant impact on their students. Finally, this study found that the measurement instrument also played a moderating role. This reminds future researchers to carefully consider measurement tools when assessing the relationship between social support and prosocial behavior. In conclusion, this study covers a wide variety of social support types and prosocial behavior types, which makes up for the limitations of the existing related meta‐analysis and helps to parse the relationship between social support and prosocial behavior more deeply, which is of guiding significance to the growth and development of individuals.

### Limitations and future directions

It should be noted that the present meta‐analysis has several limitations, which also provide directions for future research. Only gender, region, and research methods were tested in this study. Other potential moderating variables such as study methodology, family economic status, and individual education level were not examined (Andreoni et al., 2021; Kafashan et al., 2014). In future studies, we suggest further exploring the role of other potential moderators in the relationship between social support and prosocial behavior.

In addition, in some empirical studies, when researchers measured social support and prosocial behavior, only the participants themselves reported information (Oh & Roh, 2022; Zhao et al., 2020), This can lead to problems with common method deviation (CMV) (Teng & Zhang, 2022). To address the CMV issue, teachers, parents, and peers should be included as information reporters in future studies.

## CONCLUSION

To our knowledge, the current meta‐analysis is the first to explore the relationship between social support and prosocial behavior using a three‐level meta‐analysis approach. The results showed a significant positive correlation between social support and prosocial behavior, which indicated that social support was a significant predictor of prosocial behavior production. Subsequent moderating effects revealed that a number of moderating variables could explain differences in the strength of the relationship between social support and prosocial behavior: publication year, source of social support, measure of social support, and measure of prosocial behavior. Our study has important implications for understanding the role of social support in the production of prosocial behavior, but also for measures that promote the development of prosocial behavior in individuals.

## CONFLICT OF INTEREST STATEMENT

The authors declare that they have no conflicts of interest.

## ETHICS STATEMENT

This study was reviewed and approved by the Ethics Committee of the School of Education, West China Normal University.
